# Overlapping clinical manifestations of COVID-19 with endemic infectious diseases in Pakistan: A looming threat of multiple lethal combinations

**DOI:** 10.1080/20008686.2021.1873494

**Published:** 2021-01-20

**Authors:** Muhammad Suleman Rana, Muhammad Usman, Muhammad Masroor Alam, Aamer Ikram, Muhammad Salman

**Affiliations:** Department of Virology, National Institute of Health, Islamabad, Pakistan

Dear Editor,

The clinical similarity of COVID-19 with other pathogens is a challenge for the laboratory confirmation without a differential diagnosis. Here we want to present our data on endemic infectious diseases and alarming threat of multiple co-infections which is the cause of serious public health concern in Pakistan.

In Pakistan as of 13 October 2020, the toll of confirmed COVID-19 cases reached 319,848 including 6,588 deaths after its first confirmation on 26 February 2020[1].

The total population of Pakistan is more than 221.5 million people, making it the fifth largest populous country in the world. According to the 2017 census, there is a 57% increase in population during the last 19 years and around 30–40% of the population lived below the poverty line[2]. Due to overcrowded population, poverty and poor healthcare infrastructure, many infectious diseases are common in Pakistan, such as dengue, tuberculosis, (TB) malaria, measles, typhoid, hepatitis and influenza. The endemicity of these infectious diseases has been sustained in Pakistan for decades. The official number of laboratory confirmed dengue cases reported in Pakistan from 1982 to 2019 are 141547 including 844 deaths. Last year 47,120 dengue cases including 75 deaths compared to 3204 dengue cases including 2 deaths were reported in 2018 [3]. For Tuberculosis (TB), the numbers of cases are increasing and around 27,000 new cases are reported every year. According to the national TB control program data the incidence rate is 5, 25,000 per year; however, there are 3,68,589 TB patients are under treatment and 56,000, TB-related deaths occurred every year in Pakistan [4]. Malaria is another leading cause of morbidity and mortality in Pakistan. Pakistan is amongst the seven countries of the WHO Eastern Mediterranean Region sharing 98% of the total regional malaria burden and an estimated 177 million population is at high risk of malaria. It is estimated that there are 1 million suspected and 300,000 confirmed malaria cases are reported each year from Pakistan [5]. Around 50,000 deaths due to Malaria occurred every year in the country [6].

Despite the availability of a safe and effective vaccine, measles remains one of the leading causes of death among children in Pakistan. More than 8,345 measles cases were reported by the laboratory surveillance system in 2019 and 34,000 measles cases were reported during 2018 [7]. Measles accounts for around 20,000 deaths of Pakistani children each year [8]. For typhoid, there are 22,571 total cases reported during 2016–2020 and situation becomes worrisome when 16000 (70%) out of 22,571 cases were identified as extensively drug-resistance (XDR) typhoid [9]. Pakistan is among one of the first countries in the world to introduce the typhoid conjugate vaccine in 2019 in the expanded program on immunization (EPI) schedule [10]. Pakistan stands second in the world regarding the prevalence of viral hepatitis and over 18 million Pakistani are infected from viral hepatitis B and C. Approximately 150,000 deaths occurred every year and around 400 hepatitis patients died daily in Pakistan, whereas over, 250,000 new cases reported per year [11].

Influenza virus outbreaks occurred every year in Pakistan and during 2018, there were 928 suspected cases, 192 laboratory confirmed cases with 20 related deaths have been reported by national health authorities [12].

Endemic infectious diseases and newly emerging infectious diseases such as COVID-19 epidemic are expected to overlap in dengue, TB, malaria, hepatitis, measles, typhoid and influenza endemic countries. COVID-19 and endemic infectious diseases in Pakistan shared high level of similarities among the clinical and laboratory characteristics (Table 1). The clinical picture of COVID-19 often confused with these endemic infections and lead to challenges in the early diagnosis. The convergence in the symptomology of multiple pathogens can be identified through the laboratory confirmation using a battery of testing to confirm underlying infectious diseases. The co-epidemic of COVID-19 and endemic infectious in Pakistan poses multiple public health challenges and such type of multiple combinations not only complicating the diagnosis but also present a double burden on the fragile healthcare system of the country. The differential diagnosis of multiple pathogens of similar clinical nature depends upon the availability of different kits, diagnostic capacity, financial resources and technical staff as well. Early identification of COVID-19 patients co-infected with other viral or bacterial pathogens is crucial for selecting the treatment options and it will be helpful for reducing the rate of mortality.

**Table 1.** Overlapping clinical and laboratory characteristics of COVID-19 and endemic infectious diseases in Pakistan

Clinical Symptoms	COVID-19	Dengue	TB	Malaria	Measles	Typhoid	Hepatitis	Influenza
Fever	+++	+++	+++	+++	+++	+++	++	++
Headache	+++	+++	+++	++	++	++	++	++
Rash	+	+	+	+	+++	+	+	+
Myalgia	+++	+++	+	+++	++	++	++	++
Cough	+++	+	+	+	+	+	+	+++
Sore Throat	++	-	+	+	+	+	-	+
Diarrhea	++	+	+	+	+++	+++	++	+
Vomiting	+	+	++	++	+	++	+	-
Chest Pain	++	+	+++	+	+	+	-	+
**Laboratory Findings**								
Hb	↓	**N**	↓	↓	**N**	↓	**N**	**N**
ESR	↑	**N**	↑	↑	**N**	↑	**N**	↑
WBCs	↓	↓	**N**	↓	**N**	↑	↓	**N**
Neutrophils	↑	↓	↑	↑	**N**	↓	**N**	**N**
Lymphocytes	↓	↓	↑	**N**	↑	↑	**N**	↓
Platelets	↑	↓	**N**	↓	↓	↓	**N**	↓
ALT	↑	↑	↑	↑	↑	↓	↑	↑
AST	↑	↑	↑	↑	↑	↑	↑	↑
ALP	↑	↑	↑	↑	↑	↑	↑	**N**
Bilirubin	↑	↑	↑	↑	**N**	↑	↑	**N**
LDH	↑	↑	↑	↑	↑	↑	↑	↑
Total Proteins	↓	↓	↓	↑	**N**	↑	↓	**N**
Urea	↑	↑	↑	↑	**N**	**N**	**N**	↑
Creatinine	↑	↑	↑	↑	**N**	↑	**N**	**N**
Sodium	↓	↓	↓	↓	**N**	↓	↓	**N**
Potassium	↓	↓	↑	↓	**N**	↓	↑	**N**
Calcium	↓	↑	↑	↓	**N**	↑	↑	**N**
CRP	↑	↓	↑	↑	↑	↑	↓	↑

WBC = White blood cells, ALT = Alanine amino transferase, AST = Aspartate amino transferase, ALP = Alkaline phosphatase, LDH = Lactate dehydrogenase, CRP = C- Reactive protein, N = Normal

It is reported that the lymphopenia seen in majority of COVID-19 patients may increase the susceptibility to other infectious diseases and the chances of secondary infections with COVID-19 patients among fatal cases might be as high as 50% [13]. The healthcare professionals should be aware of the possibility of multiple co-infection, as the co-infection of COVID-19 with dengue, TB, malaria, hepatitis and influenza has already been reported with unfavorable outcome [14–18]. The concurrent infection of COVID-19 with other common infectious diseases should be carefully reported and follow by the active surveillance. Additional information are also required to investigate whether the patients with concurrent infection have worse outcome as compared to those mono-infected COVID-19 patients.

## Author information

Department of Virology, National Institute of Health, Park Road, Chak Shehzad, Islamabad Pakistan

Overlapping clinical and laboratory characteristics of COVID-19 and endemic infectious diseases in Pakistan
